# Effect of Chemotherapeutic Drugs on Caspase-3 Activity, as a Key Biomarker for Apoptosis in Ovarian Tumor Cell Cultured as Monolayer. A Pilot Study

**Published:** 2015

**Authors:** Ewa L Gregoraszczuk, Agnieszka Rak-Mardyła, Janusz Ryś, Jerzy Jakubowicz, Krzysztof Urbański

**Affiliations:** a *Department of Physiology and Toxicology of Reproduction, Institute of Zoology, Jagiellonian University, 30-389 Krakow, Gronostajowa 9, Poland. *; b *Department of Tumor Pathology *; c *Department of Gynecological Oncology, Center of Oncology, Maria Sklodowska-Curie Memorial Institute, Krakow, Garncarska 11, Poland.*

**Keywords:** Ovarian cancer, Monolayer cell culture, Chemotherapeutic drugs, Casapase-3 activity, Alamar Blue test

## Abstract

We aimed to develop a cost-effective and robust method to predict drug resistance in individual patients. Representative tissue fragments were obtained from tumors removed from female patients, aged 24-74 years old. The tumor tissue was taken by a histopathology’s or a surgeon under sterile conditions. Cells obtained by enzymatic dissociation from tumor after surgery, were cultured as a monolayer for 6 days. Paclitaxel, doxorubicin, carboplatin and endoxan alone or in combination were added at the beginning of culture and after 6 days, Alamar blue test was used for showing action on cell proliferation why caspase- 3 activity assays for verifying action on apoptosis. Inhibitory action on cell proliferation was noted in 2 of 12 patients tumor treated with both single and combined drugs. Using caspase-3 assay we showed that 50% of tumor cells was resistant to single chemotherapeutic drugs and 40% for combined. In 2 of 12 tumors, which did not reacted on single drugs, positive synergistic action on cell proliferation was observed in combination of D + E and C + E. This pilot study suggests: 1) monolayer culture of tumor cells, derived from individual patients, before chemotherapy could provide a suitable model for studying resistance for drugs; 2) caspase-3 activity is cheap and useful methods; 3) Alamar blue test should be taken into consideration for measuring cell proliferation.

## Introduction

Ovarian cancer is the most lethal gynecologic malignancy in the developed world (1), and there are still some debates about whether it is better to use chemotherapy before or after surgery. Currently, the preferred treatment regimen for ovarian cancer is combination chemotherapy; usually a platinum based drug, such as cisplatin or carboplatin, coupled with paclitaxel.

However, advanced ovarian cancer typically becomes chemo-refractory within approximately 2 years, and second-line treatment options do not provide a significant survival advantage (2). It has been showed previously, similar response rate between 10-40% of several agents that have been used, including retreatment with paclitaxel and carboplatin (3-6). Therefore, it is important to identify agents or drug combinations that are active in recurrent disease and can be used as salvage chemotherapy for epithelial ovarian cancer (EOC). The two most pressing problems in the management of ovarian cancer are the lack of adequate screening strategies and the chemoresistancy of recurrent disease. In part, the deficiency in diagnostic tools is due to the lack of markers for the detection of preneoplastic or early neoplastic changes in the ovarian surface epithelial cells. The wide spectrum toxic side effects of cytotoxic treatments, as well as drug resistance, are important limitations for the management of ovarian cancer and thus, methods to rapidly assess the effectiveness of treatments for individual patients are needed (7). 

Studies in cell lines do not always correlate well with results from tumor tissue, which may consist largely of non-neoplastic cells that support and modify the biology of neoplastic cells (8). Therefore, it is necessary to identify robust and rapid methods to assess the action of chemotherapeutic drugs on biopsy material or tissue taken during surgery. Ideally, the method should have the ability to determine individual patient responses to each drug. Extreme drug resistance (EDR) assays have been used to identify chemotherapy regimens that are least likely to be of clinical benefit in the treatment of EOC (9). EDR assay results do not independently predict or alter the outcomes of patients with EOC who are treated with the current standards of primary cytoreductive surgery followed by platinum and taxane combination chemotherapy. Extreme drug resistance assay results do not influence survival in women with epithelial ovarian cancer (10). EDR is methodologically similar to the thymidine incorporation assay, using metabolic incorporation of titrated thymidine to measure cell viability. 

The ability of a cancer cell to respond to a chemotherapeutic agent is believed to be due, in part, to its apoptotic capacity (11). In the present study we decided to determine the effect of single or combined chemotherapeutic drugs on proliferation using Alamar Blue test and caspase-3 activity as a key biomarker for apoptosis in ovarian cancer cells obtained from patients after surgery. 

## Experimental

*Material and methods*

*Reagents*

Hams F-10 medium, collagenase type H, antibiotic-antimycotic solution (100 ×), fetal bovine serum (FBS, heat-inactivated) and trypan blue were obtained from Sigma Chemical Co. (St. Louis, USA). Phosphate-buffered saline (PBS) was purchased from the Laboratory of Sera and Vaccines (Lublin, Poland). Paclitaxel, doxorubicin, carboplatin and endoxan were obtained from the Oncological Surgery Department of the National Cancer Institute, Cracow Branch, and Poland.

*Tumors samples*

Representative tissue fragments were obtained from tumors removed from female patients, aged 24-74 years old, who were treated in the Department of Gynecological Oncology, Cracow Center of Oncology. The characteristics of the tumors studied are depicted in the Table 1, classified according to Tavassoli *et al.* (12). Twelve, well-defined primary malignant tumors were selected for this study. These included six serous adenocarcinoma, three endometrioid adenocarcinoma, one serous adenoma of borderline malignancy, one serous cystadenoma of borderline malignancy, and one surface papillary adenocarcinoma.

**Table 1:** Characteristics of ovarian cancers studied. *classified according to Tavassoli FA, Devilee P (Eds), WHO Classification of Tumours. Pathology and Genetics of Tumours of the Breast and Female Genital Organs, IARC Press Lyon 2003.

**Case no**	**Age of patient**	Histological type of tumor*
1	64	Serous adenocarcinoma G.3
2	74	Serous adenocarcinoma G.3
3	67	Serous adenocarcinoma G.3
4	24	Serous adenoma of borderline malignancy
5	56	Serous adenocarcinoma G.3
6	72	Serous adenocarcinoma
7	47	Surface papillary adenocarcinoma
8	30	Serous cystadenoma of borderline malignancy
9	43	Endometrioid adenocarcinoma
10	63	Endometrioid adenocarcinoma G.2
11	53	Endometrioid adenocarcinoma G2
12	48	Serous adenocarcinoma

Tumor tissue was taken by a histopathology’s or surgeon under sterile conditions, and transported to the laboratory in PBS with antibiotics (100U/mL penicillin and 100ng/mL streptomycin) at 4°C. Ovarian tumor tissue was cut into small pieces and placed in Petri dishes containing PBS. Cells were obtained by enzymatic dissociation, primarily using 0.75mg/mL collagenase (Sigma Cat No. C-8051), repeated three times for 15 min at 37°C. Cells were then assessed for viability by trypan blue exclusion. Approximately 20,000–30,000 cells/well were added to each well of a 96-well plate with 200µL/well 10% (FBS) medium. Plates were then incubated at 37°C in 5% CO_2_ for 24h to allow the cells to attach. Cell culture medium was then replaced with Hams F-10 containing 5% FBS and the following drugs: paclitaxel (13.8µM), doxorubicin (2.5µM), carboplatin (13.4µM), or endoxan (19µM), either alone or in the following combinations: 1) paclitaxel plus doxorubicin; 2) paclitaxel plus carboplatin; 3) paclitaxel plus endoxan; 4) doxorubicin plus carboplatin; 5) doxorubicin plus endoxan; or 6) carboplatin plus endoxan. Drug doses were based on those of Nicolantonio *et al.* (13) and doses used during chemotherapy. The experiment was terminated after 6 days. After cell culture, cell proliferation was measured using an Alamar Blue assay. Cells were stored at -20C prior to estimation of caspase-3 activity.

**Table 2:** Percent inhibition (-) or stimulation (+) of cell proliferation by chemotherapuetic agents. Cells were treated with paclitaxel (P), carboplatin (C), doxorubicin (D), endoxan (E), paclitaxel plus doxorubicin (P + D), paclitaxel plus carboplatin (P + C), paclitaxel plus endoxan (P + E), doxorubicin plus carboplatin (D + C), doxorubicin plus endoxan (D + E), or carboplatin plus endoxan (C + E).

**Chemotherapeutic drugs**											
Patient	P	C	D	E	P+D	P+C	P+E	D+C	D+E	C+E
P1	-30	-50	-56	-54	-25	-25	-30	-50	-50	-50
P2	+40	0	0	0	+40	+30	+40	0	0	0
P3	+190	0	0	0	+110	+102	+85	10	0	0
P4	+20	0	0	0	+40	+20	+25	+10	+10	+10
P5	+40	0	0	0	+70	+50	+50	+10	+10	0
P6	+35	0	0	0	+90	+70	+80	+100	0	+60
P7	+30	+30	+10	0	+40	+20	+40	+50	0	+10
P8	+70	+10	0	0	+90	+70	+60	+10	+10	0
P9	+60	0	0	0	+70	+60	+30	0	0	+10
P10	-25	-74	-77	0	-55	-50	-27	-40	-40	-20
P11	+70	+10	+10	+10	+106	+90	+90	+20	+20	+10
P12	+102	0	0	0	+90	+100	+90	0	+20	+10

*Measurement of cell proliferation using Alamar Blue*

Alamar Blue assay (Bio Source International, USA) is designed to measure quantitatively the proliferation of various human and animals cell lines based on detection of metabolic activity. The active ingredient is resazurin (IUPAC name: 7-hydroxy-10-oxidophenoxazin-10-ium-3-one), also known as diazo-resorcinol, azoresorcin, resazoin, resazurine, which is water-soluble, stable in culture medium, is non-toxic and permeable through cell 50 years to assess cell viability and cytotoxicity in a range of biological and environmental systems and in a number of cell types including bacteria, yeast, fungi, protozoa and cultured mammalian and piscine cells. It offers several advantages over other metabolic indicators and other cytotoxicity assays (14, 15). The REDOX indicator produces a clear, stable distinct change which is easy to interpret. After 6 days of culture, Alamar Blue was aseptically added to culture wells in an amount equal to 10% of the incubation volume, and after a 5 h-incubation the absorbance of the medium was measured at 570 nm and 600 nm wavelengths using a fluorescence microplate reader (BioTek Instruments, VT, USA).

*Measurement of apoptosis by caspase-3 activity*

The caspase-3 colorimetric assay is based on the hydrolysis of the peptide substrate acetyl-Asp-Glu-Val-Asp p- nitroanilide (Ac-DEVD-pNa) by caspases-3, resulting in the release p-nitroaniline (pNA) moiety. The concentration of the pNA released from the substrate is calculated from the absorbance values at 405 nm. The comparison of the absorbance of pNA from an apoptotic sample with an uninduced control allows determination of the fold increase in caspase-3 activity. After replacing media with caspase assay buffer (50 mM HEPES, pH 7.4, 100 mM NaCl, 0.1% CHAPS, 1 mM EDTA, 10% glycerol and 10 mM DTT), cell lysates were incubated with a caspase-3 colorimetric substrate, acetyl-Asp-Glu-Val-Asp-p-nitroanilide-7-amino-4-methylcoumarin (Ac-DEVD-AMC conjugation); Sigma). Reaction mixtures were incubated at 37°C for 15 min to 1h, and the absorbance was measured at 405nm using an ELISA microplate reader (BioTek Instruments). Caspase 3, which is an effector caspase, is the most studied of mammalian caspases. Caspase 3 plays a central role in mediating nuclear apoptosis including chromatin condensation and DNA fragmentation as well as cell blebbing. Caspase 3 activity is tissue, cell type, or death stimulus specific. 

*Statistical analysis*

Each treatment was repeated three times (n = 3) in quadruplicates, and the average of the values was used for statistical calculations. Statistical analysis was performed using Stastistica 6.0 (StatSoft Inc., OK, USA). Data were analyzed by one-way analysis of variance (ANOVA) followed by the Tukey honestly significant difference (HSD) multiple range test. 

**Table 3:** Percent stimulation (+) of apoptosis by chemotherapuetic agents. Cells were treated with paclitaxel (P), carboplatin (C), doxorubicin (D), endoxan (E), paclitaxel plus doxorubicin (P + D), paclitaxel plus carboplatin (P + C), paclitaxel plus endoxan (P + E), doxorubicin plus carboplatin (D + C), doxorubicin plus endoxan (D + E), or carboplatin plus endoxan (C + E).

**Chemoteraupetic drugs**											
Patient	P	C	D	E	P+D	P+C	P+E	D+C	D+E	C+E
P1	0	0	0	0	0	0	0	0	0	0
P2	0	0	0	0	0	0	0	0	0	0
P3	0	0	0	0	0	0	0	0	0	0
P4	0	43 +	+30	+50	0	0	+35	0	+50	+60
P5	0	0	0	0	0	0	0	0	0	0
P6	0	0	0	0	0	0	0	0	0	0
P7	+80	+98	+80	+80	0	+141	+165	0	+200	+260
P8	+80	+60	+80	+85	+45	+90	+108	0	+220	+280
P9	0	0	0	0	0	+80	0	+70	+95	+70
P10	+80	0	+45	0	0	0	+60	0	0	0
P11	+195	+102	+40	+130	+190	+160	+160	0	+70	+90
P12	0	0	0	0	0	0	0	0	0	0

## Results and Discussion

**Figure 1:**
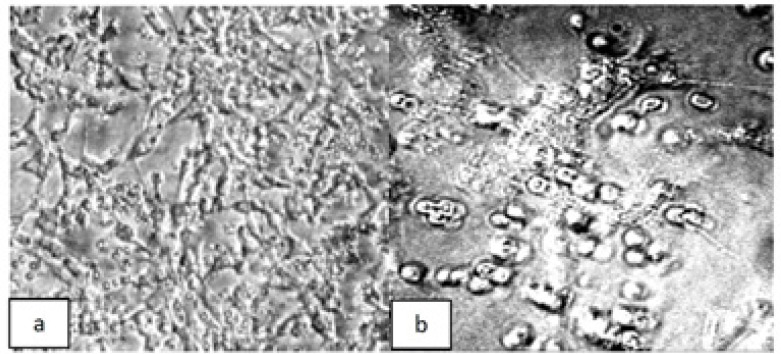
Phase contrast photomicrographs of ovarian tumor cells derived from patient 1, and cultured for 6 days in (a) control media or (b) media containing paclitaxel plus carboplatin

In the presented data we used monolayer cell cultures obtained from individual patients. Cells were exposed for 6 days to individual or combined chemotherapeutic drugs. Figure 1 shows a representative six days monolayer culture in control and drug treated cells. 

**Figure 2:**
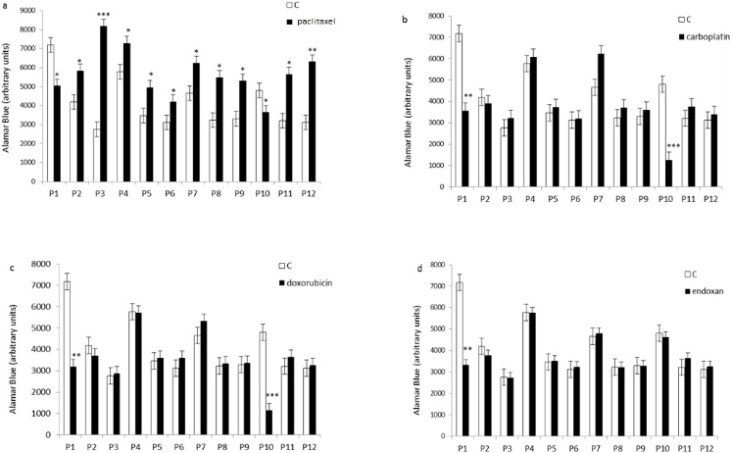
Effect of individual chemotherapeutic drugs on tumor cell proliferation. Cells derived from 12 patients (P1–12) were treated with paclitaxel (13.8µM), doxorubicin (2.5µM), carboplatin (13.4µM), or endoxan (19µM) for 6 days. Cell proliferation was measured using an Alamar Blue assay. *p < 0.05, **p < 0.01, statistically significant inhibition or stimulation of cell proliferation

**Figure 3:**
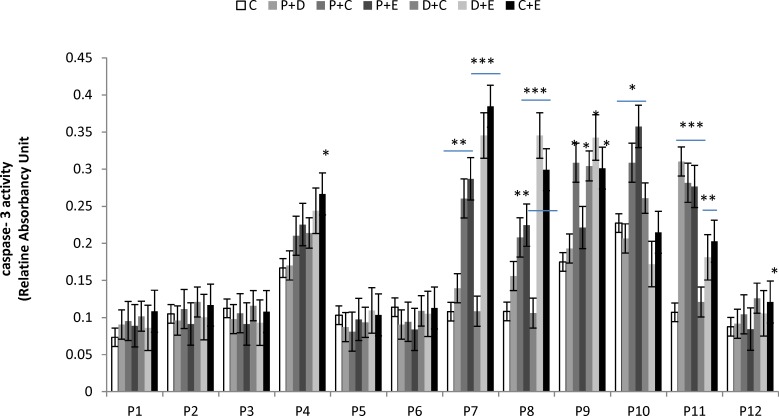
Effect of combined chemotherapeutic drugs on tumor cell proliferation. Cells were treated with paclitaxel plus doxorubicin (P + D), paclitaxel plus carboplatin (P + C), paclitaxel plus endoxan (P + E), doxorubicin plus carboplatin (D + C), doxorubicin plus endoxan (D + E), or carboplatin plus endoxan (C + E) for 6 days. Cell proliferation was measured using an Alamar Blue assay. *p < 0.05, statistically significant inhibition of cell proliferation; #p < 0.05, statistically significant stimulation of cell proliferation

It has been showed that 80% of the tumor cells were resistant to chemotherapeutic drugs. We observed inhibition of cell proliferation in two of the 12 tumor cultures treated with paclitaxel, carboplatin or doxorubicin using Alamar Blue test, and only one of the 12 cultures treated with endoxan (Figure 1). Four of the 12 cultures did not respond to the single or combined drugs. In six of the 12 tumor cell cultures, paclitaxel stimulated cell proliferation (Figure 2), and in 10 of the 12 cultures, all of the combinations containing paclitaxel stimulated cell proliferation (Figure 3). According to a new study recently published in the peer-reviewed journal Nature, chemotherapy not only promotes the growth and spread of cancer cells by damaging the healthy tissue that surrounds tumors, but it also causes cancer cells to develop full-on resistance to the popular treatment, morphing them into “super” cancer cells (16) which would explain the our findings in the case of some patients. 

**Figure 4:**
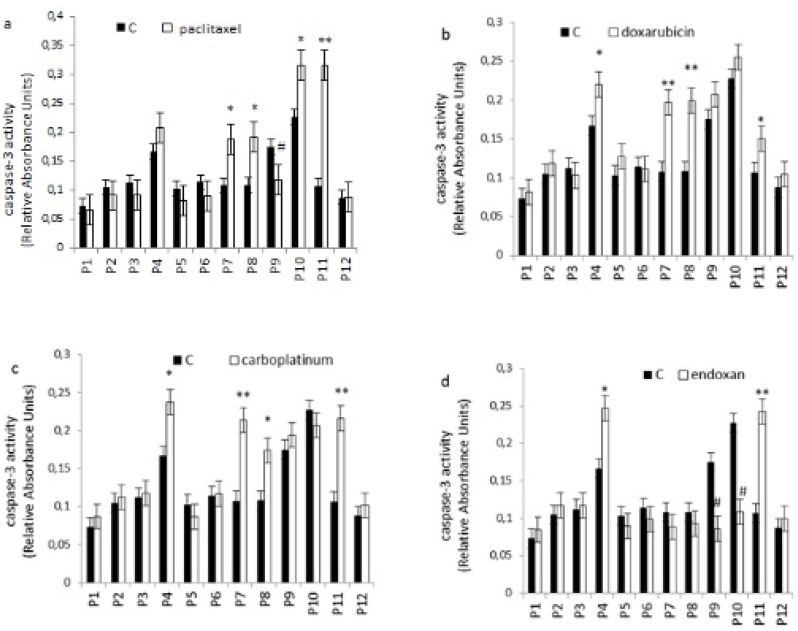
Effect of individual chemotherapeutic drugs on tumor cell apoptosis. Cells were treated with paclitaxel (13.8µM), doxorubicin (2.5µM), carboplatin (13.4µM) or endoxan (19µM) for 6 days. Apoptosis was measured using a caspase-3 assay. *p < 0.05, **p < 0.01, statistically significant stimulation of apoptosis; #p < 0.05, statistically significant inhibition of apoptosis

**Figure 5:**
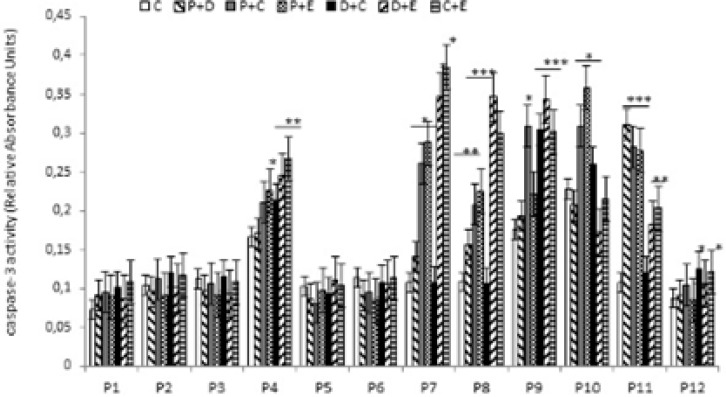
Effect of combined chemotherapeutic drugs on tumor cell apoptosis. Cells were treated with paclitaxel plus doxorubicin (P + D), paclitaxel plus carboplatin (P + C), paclitaxel plus endoxan (P + E), doxorubicin plus carboplatin (D + C), doxorubicin plus endoxan (D + E), or carboplatin plus endoxan (C + E) for 6 days. Apoptosis was measured using a caspase-3 assay. *p < 0.05, **p < 0.01, ***p < 0.001, statistically significant stimulation of caspase-3 activity

Apoptosis is programmed cell death that involves the controlled dismantling of intracellular components while avoiding inflammation and damage to surrounding cells. Apoptosis is a mode of programmed cell death that is coordinated by members of the caspases family of cysteine proteases. The caspase-3 plays a central role in the execution-phase of cell apoptosis. Several attempts have been made within the last decade to develop molecules capable of directly activating caspase-3 for use in cancer therapy. A particular target suggested for intervention has been the “safety catch” sequence present in inactive procaspase-3 (17). Using caspase-3 activity assays we noted that six of the 12 (50%) tumor cell cultures were resistant to single chemotherapeutic drugs. Caspase-3 activity in three of the 12 tumor cell culture was stimulated by all four drugs tested, whereas two of the 12 cell culture were stimulated by two or three of the drugs (Figure 4). Combination treatments had no effect on five of the 12 cell lines (40%). Two tumor cell cultures that did not respond to single drug treatments responded to two of the four combinations, and five of the 12 tumor cell cultures which were resistant to single drugs were also resistant to combination treatments (Figure 5).

The results presented are in agreement with previously published data. Chemotherapeutic drug resistance is a common feature of ovarian cancer and is a major problem given that the majority of EOCs are in an advanced stage when a diagnosis is made (18). The standard therapy for women with advanced EOC is paclitaxel, or similar, cisplatin-based combinations (19). Paclitaxel combined with cisplatin or carboplatin is a favored regimen for treating epithelial cell ovarian cancer. It produces complete disease regression in about one quarter of patients with Stage III disease, with two thirds of patients having a meaningful therapeutic response. 

 In clinical trials in 1989, paclitaxel was reported to produce partial or complete responses in 30% of patients with advanced ovarian cancer (19). Recently, the Gynecologic Oncology Group reported that the incorporation of paclitaxel into first-line therapy for ovarian cancer improved the duration of progression-free survival, and overall survival (20, 21). Di Nicolantonio *et al.* (13) have shown development of such resistance to combination chemotherapy in tumor-derived cells from matched biopsies collected from breast cancer patients before and after administration of doxorubicin-containing chemotherapy. This study suggests that up-regulation of resistance genes or down-regulation of target genes may occur rapidly in human solid tumors within days of treatment commencing, and that similar changes are present in pre- and post-chemotherapy biopsy material. The molecular processes used by each tumor appear to be linked to the drug used, but there is also heterogeneity between individual tumors, even those with the same histological type, in terms of the response pattern and magnitude to the same drugs. Adaptation to chemotherapy may explain why prediction of resistance mechanisms is difficult on the basis of tumor type alone or individual markers, and suggests that more complex predictive methods are required to improve the response rates to chemotherapy. 

Our data are also in agreement with recent published data by Pitakkarnkul *et al.* (4), which showed that the overall response rate to paclitaxel treatment for 41 patients was 41.5%, but was only 12.5% for patients with refractory or platinum-resistant cancer, and was 48.5% for patients with platinum-sensitive disease. Stable disease was demonstrated in 17% of patients while progressive disease was apparent in 41.5%. The same study also showed that neutropenia, neuropathy and alopecia are common side effects. Sharma* et al.* (3) evaluated clinical responses following ATP-tumor chemo sensitivity assay-directed salvage chemotherapy in 44 patients with advanced ovarian cancer. Of the 44 patients, 18 were resistant to platinum-based treatments and 31 were resistant to cisplatin (3). A recent paper by *van der Burg et al.* (5) demonstrated that weekly paclitaxel/carboplatin followed by 3-weekly cycles is highly effective against platinum-resistant EOC. After six 3-weekly cycles, 51% of the platinum-resistant patients had a response, and 77% received clinical benefits with relief of symptoms. Furthermore, *Tan et al*. (6) recently investigated the efficacy of paclitaxel in 26 patients with BRCA1- and BRCA2-mutated ovarian cancers, producing a response rate to paclitaxel monotherapy of 46%. 

## Conclusion

Activation of the caspase-3 pathway is a hallmark of apoptosis and can be used in cellular assays to quantify activators and inhibitors of the “death cascade. Our data suggest that simple, monolayer cultures of tumor cells and caspase-3 assays as a key biomarker for apoptosis are a relatively cheap and fast method taken before chemotherapy. This representative apoptosis assay can be used in an automated way and is amenable to development of high-throughput applications. However, the effective response of the patients, from whom cell cultures were derived, to front line chemotherapy and if the *in-vitro* assay will really predictive of response.
